# Microdistribution of the resistance of malaria vectors to deltamethrin in the region of Plateau (southeastern Benin) in preparation for an assessment of the impact of resistance on the effectiveness of Long Lasting Insecticidal Nets (LLINs)

**DOI:** 10.1186/1471-2334-14-103

**Published:** 2014-02-25

**Authors:** Arthur Sovi, Innocent Djègbè, Lawal Soumanou, Filémon Tokponnon, Virgile Gnanguenon, Roseric Azondékon, Frédéric Oké-Agbo, Mariam Okè, Alioun Adéchoubou, Achille Massougbodji, Vincent Corbel, Martin Akogbéto

**Affiliations:** 1 Centre de Recherche Entomologique de Cotonou, Cotonou, Benin; 2 International Institute of Tropical Agriculture, Calavi, Bénin; 3 Programme Nationale de Lutte contre le Paludisme, Cotonou, Benin; 4 University of Massachusetts Amherst, Amherst, USA; 5 Faculté des Sciences de la Santé de l’Université d’Abomey-Calavi, Cotonou, Benin; 6 Institut de Recherche pour le Développement (IRD), Maladies Infectieuses et Vecteurs, Ecologie, Génétique, Evolution et Contrôle (MIVEGEC, IRD 224-CNRS 5290 UM1-UM2), Montpellier, France; 7 Department of Entomology, Faculty of Agriculture, Kasetsart University, Bangkok, Thailand

**Keywords:** *An. gambiae s.s*, Deltamethrin, Insecticide resistance, *kdr* mutation, Plateau, Benin

## Abstract

**Background:**

This study aims to research two areas, one with a resistant and the other with a susceptible profile of *An. gambiae* to deltamethrin in the region of Plateau (southern Benin). In each area, eight localities were sought. Both areas were needed for the assessment of the impact of malaria vector resistance to pyrethroids on the effectiveness of Long Lasting Insecticidal Nets (LLINs). The susceptible area of *An. gambiae* to deltamethrin was used as a control.

**Methods:**

In total, 119 localities in the region of Plateau were screened by sampling *An. gambiae s.l* larvae. Female mosquitoes resulting from these larvae were exposed to 0.05% deltamethrin following WHO standards. PCR was used to identify species and molecular forms of the dead and alive mosquitoes. Finally, we identified *kdr* mutations (1014 F and1014S) using the HOLA technique.

**Results:**

Fifty-six out of 119 prospected localities tested positive for *Anopheles gambae s.l* breeding sites. The results showed that *An. gambiae* was resistant to deltamethrin in 39 localities and susceptible in only 2 localities; resistance to deltamethrin was suspected in 15 localities. The HOLA technique confirmed the presence of *kdr* 1014 F mutation and the absence of *kdr* 1014S mutation. The *kdr* 1014 F mutation was found in both M and S molecular forms at relatively high frequencies therefore confirming the susceptibility tests.

**Conclusion:**

We were unable to identify the eight susceptible areas due to the overall resistance of *An. gambiae* to deltamethrin in the region of Plateau. To implement the study, we kept two areas, one with high resistance (R^+++^) and the other with low resistance (R^+^) of *An. gambiae* to deltamethrin.

## Background

Benin, like many other African countries, has based its vector control strategy on two major interventions: universal access to Long Lasting Insecticidal Nets (LLINs) and Indoor Residual Spray (IRS). Thus, in late July 2011, a large scale distribution of LLINs had been conducted by Benin National Malaria Control Program (NMCP) with the support of the World Bank, the US President’s Malaria Initiative (PMI) and the World Health Organization (WHO). Another distribution campaign had already been conducted in October 2007 to ensure a partial and selective coverage in two types of LLINs, PermaNet® and OlysetNet® respectively impregnated with deltamethrin and permethrin. A national survey assessing the 2007 campaign revealed that 56.3% of children under 5 years and 54.8% of pregnant women slept under a LLIN the night before the survey [1]. The current study carried out between April and early July 2011 had taken place between the two aforementioned distribution campaigns.

Insecticide resistance has become widely distributed in Western [2-5], Eastern [6], Central [7] and Southern Africa [8]. This could be a serious obstacle to the effectiveness of LLINs. This raises an important question within National Malaria Control Programmes (NMCPs): should we continue to promote LLINs? The question has been explored by N’Guessan *et al.*[9] who demonstrated a decrease in the effectiveness of LLINs and lambdacyhalothrin IRS in an area of high resistance of *Anopheles gambiae* in Southern Benin. They compared the effectiveness of mosquito nets in two areas, one area with resistant *An. gambiae* populations to pyrethroids and one other where *An. gambiae* is susceptible to pyrethroids. However, the study was conducted on an experimental scale using experimental huts. It is therefore difficult to predict what would happen at a community level.

For that reason, a large scale study investigating the impact of malaria vector resistance to pyrethroids on the effectiveness of LLINs was initiated in late July 2011. In the context of universal access to LLINs, the implementation of such an impact study requires the identification of two areas: one area where *An. gambiae* was highly resistant to pyrethroids, and one other where *An. gambiae* was still susceptible to pyrethroids. The latter area would serve as a control area. The main objective of this article is the determination of these two areas. This will allow the comparison of the effectiveness of LLINs between the two areas.

The results of a previous study conducted in forty districts in Southern Benin revealed that the entire region is covered by pyrethroids resistant populations of *An. gambiae*[5]. However, a few pockets of susceptible populations of *An. gambiae* has been reported in two districts (Ifangni and Adja-wèrè) located in the region of Plateau [5]. This is why we selected this region for the implementation of our impact study. However, we decided to randomly implement the study in 16 localities (8 localities constituting the resistant area and 8 others representing the susceptible area). We then multiplied larval prospections and susceptibility tests in many localities across four districts within the region of Plateau. Unfortunately, we were unable to identify the 8 localities of high susceptibility of *An. gambiae* as previously expected. We then decided to replace the susceptible area by an area where *An. gambiae* has a low resistance [low resistant area (R^+^ area) vs high resistant (R^+++^ area)].

Regarding the susceptibility tests, we had the choice between deltamethrin and permethrin, two insecticides used to impregnate PermaNet® and OlysetNet®. We ended up choosing deltamethrin. This choice has been motivated by evidence suggesting a wide distribution of high vector resistance to permethrin in Southern Benin [5]. Under these conditions, we believed that the probability to identify localities where *An. gambiae* is susceptible to permethrin is low.

From April to early July 2011, we conducted several field missions across four districts in the region of Plateau. Results from the susceptibility tests helped classify the localities between the two resistant (R^+^ and R^+++^) areas. In both R^+^ and R^+++^ areas, a study of malaria transmission and vector behavior had been implemented.

## Methods

### Study area

The study was conducted in the region of Plateau in the south-east of Benin and specifically in the districts of Ifangni, Sakété, Pobè and Kétou. This region has an area of 3,264 km^2^, with a total population of 407,116 inhabitants [10]. Fifty-six localities which tested positive for *An. gambiae* larvae are mostly rural and represent 31.3% (56/179) of the localities of the four districts to which they belong, which total area is 2,849 km^2^[10]. The climate in the Plateau region is Guinean with two rainy seasons and two dry seasons. The region records an annual rainfall between 800 mm and 1200 mm in its western part and between 1000 mm and 1400 mm in its eastern part. The cropping system is characterized by the practice of two annual growing seasons associated with rainfall patterns. Corn crop is predominant. The whole area is strewn with swamps. These swamps are used for the production of off-season crops and the installation of ground farms for various species. Different economic activities take place in the region of Plateau because of the opportunities offered by the natural environment but also its closeness to Nigeria, which has a polarizing action in the region. The region of Plateau abounds with farmers and traders.

### Collection and breeding of larvae and pupae of Anopheles mosquitoes

From April to early July 2011, 119 localities were prospected in all four districts, of which 56 tested positive for *Anopheles* breeding sites. Localities which tested positive were geo-referenced using a Global Positionning System (GPS).

Larvae and pupae of *An. gambiae* were collected in collections of water using the "dipping" technique [11]. This technique consists of collecting larvae and pupae, using a simple ladle, from positive breeding sites. Larvae and pupae were kept separately in labeled bins and taken to the insectary of the “Centre de Recherche Entomologique de Cotonou” (CREC) for rearing. After emergence, adults of 2 to 5 days old were used for susceptibility testing in the laboratory.

### Susceptibility tests

Susceptibility tests were performed according to the WHO susceptibility tube-test with unfed female *Anopheles*, aged 2 to 5 days. These tests were performed with paper impregnated with deltamethrin at the diagnostic dose of 0.05%.

Batches of 25 female mosquitoes aged 2 to 5 days were added to each tube carpeted with deltamethrin impregnated paper for 60 minutes. Batches exposed to untreated papers were used as control. Mosquitoes of the susceptible Kisumu strain were exposed to deltamethrin (0.05%) treated filter papers. The number of field mosquitoes knocked down as a result of the insecticide effect was recorded every ten minutes during the exposure time period. After 60 minutes of exposure, mosquitoes were transferred to the observation tubes and fed with a 10% honey solution and kept under observation for 24 hours. At the end of the observation time, mortality rates were determined. These rates were construed in accordance with the recommended criteria by WHO [12]. The resistance status was determined based on the following criteria:

–Mortality > 97%: susceptible *Anopheles* population.

–Mortality 80 – 97%: suspected resistance in the *Anopheles* population.

–Mortality < 80%: resistant *Anopheles* population.

Given the small number of localities where *An. gambiae* was susceptible to deltamethrin, we decided to define R^+^ localities as all localities where *Anopheles* mortality was greater than or equal to 80%. All localities with mortality rates below 80% were considered highly resistant (R^+++^ localities).

We did not choose the 80% cutoff at random to separate both areas. This criterion was chosen according to the WHO standard to identify highly resistant mosquitoes [12]. We considered suspected resistant mosquitoes and susceptible ones [12] as lowly resistant.

After susceptibility testing, mosquitoes were kept on silicagel at −20°C for molecular characterization.

### Selection of the localities into R^+^ and R^+++^ areas

After testing for susceptibility, we randomly selected the sixteen localities for the implementation of our impact study at both defined resistance levels (R^+^ and R^+++^) in order to avoid selection bias.

### Molecular characterization of *An. gambiae*, PCR species, molecular forms and kdr 1014 F and 1014S

For each locality, 22–49 females *Anopheles* were analyzed by PCR according to the protocol described by Scott *et al.*[13]. Identification of *Anopheles* species was made according to the protocol described by Favia *et al.*[14]. For each locality, DNA extracts from *An. gambiae* allowed us to look at the simultaneous presence of *kdr* mutation 1014 F and 1014S as recommended by Lynd *et al.*[15].

### Mapping of the resistance

The geographical coordinates of the localities where larvae were collected from were recorded by GPS and projected onto a map of the region of Plateau. The three levels of susceptibility of *Anopheles* to deltamethrin (0.05%) according to the criteria of the WHO [12] (sensitive, resistant and suspected resistance) were shown on the map. Overall, 56 localities were mapped.

### Statistical analysis

The calculation of mortality rates was performed using MS Excel spreadsheet. The chi-square test of comparison of proportions was used to compare mortality rates within localities of the same district and within the two defined areas (R^+^ and R^+++^). Knockdown times (KdT50 and KdT95) were determined by logistic regression with probit link. We used the delta-method to determine the 95% Confidence intervals of the KdTs for the two areas. The variation in *kdr* frequency between the two areas, and between both M and S forms of *An. gambiae* was assessed via a logistic regression [16]. The same statistical method was used to assess the spatial variation in resistance levels within districts. The analysis of deviance penalized by the dispersion parameter [17] was used to assess the relevance of the variability.

All analyses were performed with R-2.15.2 statistical software [18].

## Results

### Deltamethrin-induced mortalities in 56 localities

The *An. gambiae* Kisumu reference strain was susceptible to deltamethrin (0.05%), showing 100% mortality.

Mortality rates obtained after exposing *An. gambiae* wild populations to deltamethrin (0.05%) varied between 20% and 100%.

In Ifangni, mortality rates ranged from 20% in Gblo-gblo and Djègou-djègui to 100% in Zihan. Out of 19 localities, *An. gambiae* was found susceptible only in 2 localities (Zihan and Ko-Aïdjèdo). Suspected resistance was observed in 10 localities (Table 1).

**Table 1:** **Resistance status of *An. gambiae s.l *** **populations to deltamethrin in the localities of Ifangni and Sakété**

**Districts**	**Localities**	**Total (N)**	**KdT50 (min)**	**KdT95 (min)**	**Dead (N)**	**Mortality (%)**
*Ifangni*	Lokossa*	44	38.9	74.0	36	82^a^
	Ko-dogba*	48	36.1	68,6	42	87^ad^
	Zihan*	50	60.0	114.0	50	100
	Baoudjo	89	40.5	77.0	72	81^a^
	Gbédji	88	100.0	190.0	68	77^a^
	Igolo	22	111.1	211.1	17	77^a^
	Araromi*	39	41.6	79.2	33	85^ad^
	Itassoumba*	66	39.2	74.5	59	89^ad^
	Ko-koumolou*	48	32.6	62.0	40	83^a^
	Itakpako*	131	36.6	69.5	105	80^a^
	Banigbé Centre	97	42.3	80.3	32	33^bc^
	Tchaada	22	93.8	178.1	7	32^bc^
	Daagbe*	40	31.6	60.0	33	82^a^
	Akadja	123	38.5	73.1	62	50^b^
	Gblo-gblo	45	103.4	196.5	9	20^c^
	Ko-aïdjedo*	92	31.2	59.4	90	98^d^
	Zoungodo*	62	43.5	82.6	52	84^a^
	Djegoun-djègui	40	75.0	142.5	8	20^c^
	Ketougbekon*	147	35.7	67.8	121	82^b^
*Sakété*	Ilakofadji	44	31.6	60.0	30	68^a^
	Dagbao	45	32.2	61.3	33	73^a^
	Itadjèbou	34	42.8	81.4	21	62^abc^
	Djohounkollé*	73	39.4	75.0	59	81^a^
	Igbo-abikou	65	115.4	219.2	25	38^b^
	Igbola	38	96.7	183.8	19	50^ab^
	Alabansa	30	150.0	285.0	12	40^bc^
	Iwaï*	43	37.0	70,3	37	86^a^
	Ikemon	86	71.4	135.7	40	46^bc^
	Idiagbola	40	52.6	100.0	17	43^bc^
	Yoko Centre	40	31	58.7	30	75^a^
	Idi*	31	62.5	118.7	30	97^d^

^a, b, c, ab, ac, d^The mortality rates with the different superscript in the same district are statistically different (p < 0.05).

*The localities with an asterisk are lowly resistant and those without an asterisk are highly resistant.

In Sakété, *An. gambiae* was not susceptible. In Iwaï, Idi and Djohounkollé suspected resistance was noted (Table 1).

In the district of Pobè, mortality of *An. gambiae* mosquitoes ranged from 45% in Obanigbé to 85% in Agbarou. *An. gambiae* proved resistant in all localities except Agbarou where suspected resistance was observed (Table 2).

**Table 2:** **Resistance status of *An. gambiae s.l *** **populations to deltamethrin in the localities of Pobè and Kétou**

**Districts**	**Localities**	**Total (N)**	**KdT50 (min)**	**KdT95 (min)**	**Dead (N)**	**Mortality (%)**
*Pobè*	Igbo-okpa	79	85.7	162.8	58	73^ac^
	Okeita	101	47.6	90.5	78	77^ac^
	Obanigbé	40	57.7	109.6	18	45^b^
	Agbarou*	55	66.6	126.6	47	85^a^
	Issaba	111	100	190	68	61^c^
	Okoofi 2	54	96.7	183.8	24	44^b^
	Illekpa	39	375	712.5	26	67^c^
	Osoumou 2	48	53.6	101.8	34	71^c^
	Osoumou 1	70	142.9	271.4	44	63^c^
	Onigbolo	90	52.6	100	66	73^ac^
*Kétou*	Alakouta	36	68.2	129.5	24	67^abc^
	Okpometa	57	81.1	154	23	41^a^
	Mowodani	52	68.2	129.5	31	60^ab^
	Idena 2	179	39	74	81	45^a^
	Kpankoun	70	65.2	124	37	53^ab^
	Okéola	49	66.6	126.6	24	48^a^
	Odokoto	95	33.7	64	51	54^ab^
	Igui-olou	100	49.2	93.4	72	72^bc^
	Adjozoume*	100	33.3	63.3	83	83^c^
	Idena 3	80	68.6	129.5	39	49^a^
	Oloumou	94	50.8	96.6	48	51^ab^
	Kouhoudou	60	47.6	90.5	37	62^ab^
	Obatedo	95	43.5	82.6	74	78^bc^
	Atchoubi 1	100	33	62.6	77	77^bc^
	Atchoubi 2	55	61.2	116.3	35	64^bc^

^a, b, c, ab, ac^The mortality rates with the different superscript in the same district are statistically different (p < 0.05).

The localities with an asterisk are lowly resistant and those without an asterisk are highly resistant.

In Kétou, the lowest mortality rate of *An. gambiae* (67%) was obtained in Alakouta versus 83% in Adjozounmè. Out of a total of 15 localities evaluated, suspected resistance was noted only in Adjozounmè, while full resistance was observed in all other localities (Table 2).

The lowest KdT50 (31 minutes) was obtained in Yoko Centre (district of Sakété) versus 375 minutes in Illékpa (district of Pobè) (Tables 1 and 2).

Overall, across the 56 localities of the four districts, *An. gambiae* was resistant to deltamethrin in 39 localities. However, susceptibility was noted in *An. gambiae* mosquitoes from 2 localities of the district of Ifangni. Resistance of *An. gambiae* to deltamethrin (0.05%) was suspected in 15 localities (Tables 1 and 2).

### Categorization of localities as R^+^ and R^+++^ across the four districts

Overall, out of the 56 localities, 16 were categorized as R^+^ and 40 as R^+++^ based on the mortality rates observed (Tables 1 and 2). Out of the 16 R^+^ localities, 11 including the two susceptible ones were located in the district of Ifangni, 3 in Sakété, 1 in Pobè and 1 in Kétou (Tables 1 and 2). Localities classified as R^+++^ were distributed as follows: 8 in Ifangni, 9 in Sakété, 9 in Pobè and 14 in Kétou (Tables 1 and 2).

Based on these results, 8 localities (Itakpako, Araromi, Ko-koumolou, Djohounkollé, Ko-Aïdjèdo, Lokossa, Kétougbékon and Iwaï) were randomly selected in the R^+^ area and 8 other (Mowodani, Banigbé, Okoofi 2, Ikèmon, Akadja, Idéna 2, Igbola and Tchaada) in the R^+++^ area for the implementation of our impact assessment study.

Mortality rates were 84% (516/617) in the R^+^ area made of 8 R^+^ localities versus 45% (296/651) for the R^+++^ area composed of 8 R^+++^ localities (p <0.0001). Statistical analysis also showed that the risk that mosquitoes die in contact with deltamethrin was six times higher in the R^+^ area than in the R^+++^ area (OR = 0.15, 95% CI: 0.10-0.23, p < 0.0001). In addition, the KdT95 was 67 minutes (95% CI: 52.80-86.21) in R^+^ area versus 93.4 minutes (95% CI: 87.06-113.93) in the R^+++^ area (p < 0.05).

### Molecular characterization of mosquitoes from 16 selected localities (8 R^+^ and 8 R^+++^)

#### Identification of species and forms of An. gambiae complex

After testing for susceptibility, Polymerase Chain Reaction (PCR) was used to identify species of *An. gambiae s.l* complex, and the identification of the different forms of each *An. gambiae s.s* species. This characterization was carried out on the collected mosquitoes from the 16 selected localities.

**Figure 1:**
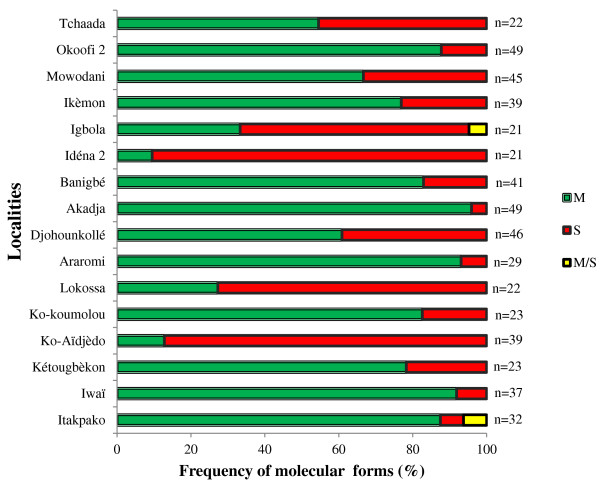
**Distribution of *An. gambiae s.s. *** **molecular forms per locality.**

The results show that out of the 506 specimens of *An. gambiae s.l* evaluated in all 16 localities, only *An. gambiae s.s* was found in all localities. Both molecular forms M and S of *An. gambiae s.s* were found in sympatry in all the 16 localities. Overall, 66.8% (338/506) of the mosquitoes belonged to the form M and 32.6% (165/506) to the form S. The proportion related to one or the other form varies depending on the locality (Figure 1). *An. gambiae s.s* form M was predominant in most localities except Lokossa and Ko-Aidjèdo in Ifangni, Igbola in Sakété and Idéna 2 in Kétou. Both forms were found in similar proportions in Tchaada (12 *An. gambiae s.s* form M out of 22).

Some hybrid individuals M/S (0.6%) were also found in Itakpako (district of Ifangni) and Igbola (district of Sakété).

#### Detection of the kdr mutation in the molecular forms (M and S) of An. gambiae s.s

The 1014S *kdr* mutation originally from East Africa was absent in 497 mosquitoes tested. However, the 1014 F *kdr* mutation was detected in the four districts of the region of Plateau. The 1014 F *kdr* mutation was found in both M and S molecular forms of *An. gambiae s.s* but at variable frequencies depending on the locality. Indeed, the frequency of the *kdr* mutation varies between 0.50 and 0.93 in the form M and between 0.45 and 0.92 in the form S (Table 3). The small number of characterized mosquitoes in some localities did not allow for accurate estimation of the frequency of this mutation. These localities are Tchaada and Lokossa in the district of Ifangni and Igbola in the district of Sakété.

**Table 3:** **Frequencies of 1014 F *kdr *** **mutation within *An. gambiae s.s. *** **molecular forms**

**Localities/Areas**	**Molecular forms**	**N**	**SS**	**RS**	**RR**	**F( *kdr *)**
*R*^*+*^*localities*						
Itakpako	M	27	0	17	10	0.69
	S	2	0	2	0	0.50
Araromi	M	24	3	18	3	0.50
	S	2	0	1	1	0.75
Ko-koumolou	M	19	1	16	2	0.53
	S	3	0	3	0	0.50
Djohounkollé	M	26	2	10	14	0.73
	S	17	0	3	14	0.91
Ko-Aïdjedo	M	5	0	4	1	0.60
	S	28	3	16	9	0.61
Lokossa	M	6	0	2	4	0.83
	S	16	0	8	8	0.75
Ketougbekon	M	15	2	6	7	0.67
	S	4	1	3	0	0.38
Iwaï	M	30	0	4	26	0.93
	S	3	0	1	2	0.83
*R*^*+*^*area*	M + S	227	12	114	101	0.70
*R*^*+++*^*localities*						
Mowodani	M	28	0	7	21	0.88
	S	12	1	4	7	0.75
Banigbe	M	28	0	10	18	0.82
	S	5	2	1	2	0.50
Okoofi2	M	43	0	12	31	0.86
	S	6	0	1	5	0.92
Ikemon	M	29	0	7	22	0.88
	S	9	0	4	5	0.78
Akadja	M	46	1	29	16	0.66
	S	2	0	2	0	0.50
Idena2	M	2	0	2	0	0.50
	S	19	2	17	0	0.44
Igbola	M	7	0	6	1	0.57
	S	12	0	8	4	0.66
Tchaada	M	11	0	9	2	0.59
	S	10	0	8	2	0.60
*R*^*+++*^*area*	M + S	269	6	127	136	0.74
*Total [(R*^*+*^*and R*^*+++*^*) areas]*	M	346	9	159	178	0.74
	S	150	9	82	59	0.67

By combining data from the two areas (R^+^ and R^+++^), the *kdr* frequency was similar within both molecular forms: M [F(*kdr*) = 0.74] and S [F(*kdr*) = 0.67] with p = 0.17 (Table 3). Furthermore, the aggregated data from the two molecular forms yielded a *kdr* gene frequency estimated at 0.70 in the R^+^ area versus 0.74 in the R^+++^ area. There is no difference between the frequency of the *kdr* mutation between the two areas (p = 0.13).

### Mapping of the resistance of *An. gambiae* to deltamethrin

**Figure 2:**
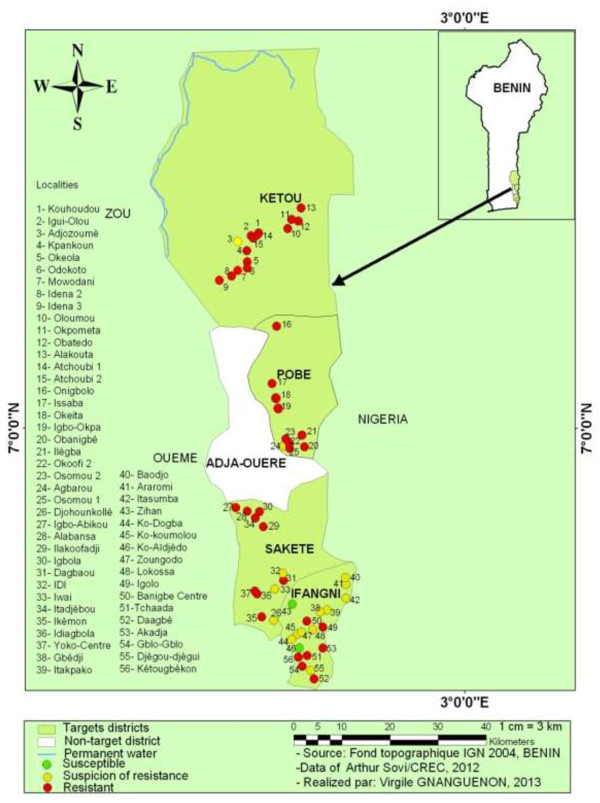
**Map showing the distribution of *An. gambiae *** **resistance to deltamethrin in the Plateau region.**

Various resistance levels to deltamethrin were observed based on the WHO criteria [12]. Figure 2 illustrates the mapping of the resistance of *An. gambiae* to deltamethrin in the prospected localities. Overall, the map shows the profile of *An. gambiae* resistance to deltamethrin in the region of Plateau in 2011. A concentration of localities where *An. gambiae* was susceptible or suspected resistance to deltamethrin is distinguishable in the district of Ifangni. On the other hand, *An. gambiae* was resistant in most localities in the districts of Sakété, Pobè and Kétou.

Table 4 shows the results of logistic regression analysis that was performed to assess differences in the level of resistance between the districts. In general, although the district of Ifangni was composed of localities where *An. gambiae* was susceptible or suspected resistant, it is clear from this analysis that the level of resistance has not been a significant variation within the four districts [p (LR-test) = 0.22].

**Table 4:** **Spatial variation of the resistance level of *An. gambiae *** **to deltamethrin within districts**

**Districts**	**Total**	**Dead (N)**	**Dead (%)**	**Coef**	**OR**	**95% CI**	**p (Wald test)**	**p (LR-test)**
Ifangni	1293	936	72.39	0.00	1.00	-	-	0.22
Sakété	569	353	62.04	−0.56	0.57	[0.29-1.14]	0.11	
Pobè	687	463	67.39	−0.24	0.79	[0.41-1.51]	0.47	
Ketou	1222	736	60.23	−0.51	0.60	[0.35-1.04]	0.06	

## Discussion

Our study shows a widespread resistance of *An. gambiae* to deltamethrin in the Plateau region, southeastern Benin. Taking into account the KdTs, the effect of deltamethrin on *An. gambiae* mosquitoes was not uniform across the various localities. Resistance with deltamethrin was associated with relatively high *kdr* frequencies found in both M and S form of *An. gambiae s.s.*

Out of the 56 localities explored, *An. gambiae* was found susceptible in only two localities (Ko-Aidjèdo and Zihan). These results show a wide distribution of deltamethrin resistance as previously reported by Padonou *et al.*[19] in certain localities of Ouéme, a department near Plateau region. The results also confirm the rapid expansion of pyrethroid resistance in natural populations of *An. gambiae* in Africa [20] and particularly in Benin [21-23]. However, the small numbers of mosquitoes exposed to deltamethrin in some localities did not allow accurate estimations of the mortality rates. This constitutes a major limitation for our study.

Previous studies demonstrated that malaria vector resistance to pyrethroids might be related to an extensive and massive use of LLINs [24,25]. Insecticide molecules at the surface of LLINs might exert a lethal effect on susceptible mosquitoes, therefore selecting for resistant mosquitoes that reproduce in natural populations. In the department of Ouémé for instance, Padonou *et al. *[19] reported an increase in the resistance of *An. gambiae* to deltamethrin following a mass distribution campaign of LLINs. It is then possible that the resistance level has increased very quickly in the region of Plateau after the selective distribution campaign of LLINs in 2007. Several other factors such as the domestic use of pyrethroids [3] and water run off loaded with insecticide particles from the North of Benin where pyrethroids were massively and uncontrollably used to control cotton pests [26] could also explained the resistance of *An. gambiae* to deltamethrin in the localities of the region of Plateau. The insecticides particles contained in the water could exert a selection pressure on the larvae of *An. gambiae*[27].

Our data surprisingly reveals a low resistance of *An. gambiae* to deltamethrin in the district of Ifangni. Nonetheless, we did not observe any spatial variation in the level of resistance across the four districts (p = 0.22). This suggests a similar selection pressure on *An. gambiae s.l* across the four districts. Therefore, other reasons, still unknown to our knowledge, may explain the low levels of resistance of *Anopheles* population to deltamethrin in the district of Ifangni. Further studies must be undertaken to understand the real causes of the low resistance of *An. gambiae* in this district.

PCR analysis reveals that *An. gambiae s.s* was the only species of the *An. gambiae s.l.* complex encountered in all 16 selected localities. The absence of *An. melas* could be explained by the fact that the larvae were all collected from small freshwater pools. This is understandable since *An. melas* larvae are mainly found in brackish water ponds [28]. Similarly, *An. arabiensis* was not present even though it has already been reported in Central Benin [23].

Regarding the molecular forms, the relative dominance of one form over the other could be explained by the presence of specific breeding sites to one or the other molecular forms [29]. Globally, in both areas (R^+^ and R^+++^), 66.8% of the mosquitoes were of M form, 32.6% of S form and 0.6% of the hybrid M/S. This contradicts Yadouléton *et al.*[5] who reported the absence of the form S of *An. gambiae s.s* in the region of Plateau. In fact the presence of the form S in significant proportions in 2011 in that region could be associated to the rainfall pattern and the rapid infiltration of water into the soil allowing the formation of favorable temporary breeding sites for the development of form S of *An. gambiae s.s*.

*Kdr* mutation 1014 F has been found in M as well S form of *An. gambiae s.s*, but at variable frequencies depending on the locality. Djènontin *et al.*[30] reported a higher frequency of *kdr* mutation in the form S than in the form M of *An. gambiae s.s* in Ouidah-Kpomassè-Tori Bossito area between October and December 2007. The same trend has been reported by Diabaté *et al.*[31] and Dabiré *et al.*[32] in the Kou valley in Burkina-Faso and by Dabiré *et al.*[33] in Guinea-Bissau. However, for our study, data from both areas (R^+^ and R^+++^) revealed a similar *kdr* frequency between the two molecular forms (p = 0.17). This suggests that the selection pressure exerted on the two molecular forms in the natural environment did not differ significantly across the region.

We did not find the 1014S *kdr* mutation originally from East Africa [34]. However, previous studies have indicated that this mutation was present in Benin [23] and was expanding to the north and center of Benin in *An. gambiae*. It is therefore important to extend the surveillance of *kdr*1014S in the region of Plateau.

Although a significant difference in mortality rates was found between both areas (R^+^ and R^+++^) (p < 0.0001), no difference was observed between the frequencies of *kdr* gene in both areas (p = 0.13). In addition, KdT95 was higher in R^+++^ area than in R^+^ area (p < 0.05). These findings suggest the involvement of other resistance mechanisms in addition to the *kdr* mutation in the mosquitoes from the R^+++^ area.

Recent studies have also reported the involvement of certain metabolic enzymes in the resistance of malaria vectors to pyrethroids in Africa [35] and in several other regions in Benin [36,37]. For example, an overexpression of CYP6M2 and CYP6P3 genes, involved in the metabolism of pyrethroids, has been reported in resistant populations of *An. gambiae* in Porto Novo [36]. Therefore, we believe that the role of metabolic resistance in the region of Plateau deserves further scrutiny.

From the results recorded from the susceptibility tests, we were able to map the distribution of the resistance of *An. gambiae* to deltamethrin. Such a map was necessary since it provides a picture of the availability of localities where *An. gambiae* was still susceptible to deltamethrin. In addition, this map could be used as an important tool to monitor the dynamics of the resistance of *An. gambiae* to pyrethroids. Moreover, given that our research activities were carried out in collaboration with Benin NMCP, this map should allow Benin NMCP to accordingly adapt its strategy of malaria prevention.

Given the extensive use of LLINs inside houses at this time, it is possible that highly resistant mosquitoes would tend to feed inside more than low resistant *Anopheles* mosquitoes. The deterrence effect of LLINs is likely to be more effective on low resistant *Anopheles* population. If this was verified, the likelihood for highly resistant mosquitoes to transmit malaria parasites would be higher than that of lowly resistant ones. If this assumption was true, it would confirm that vector resistance to insecticides is a major concern to the operational effectiveness of the LLINs distributed in 2011 [38].

## Conclusion

The results of this study show a wide distribution of the resistance of malaria vectors to deltamethrin in the region of Plateau. *An. gambiae* was found susceptible in only two locations in the district Ifangni. Two populations of *An. gambiae s.s.* were encountered in the region: *An. gambiae s.s* form M and *An. gambiae s.s* form S. Both molecular forms were resistant to pyrethroids. Besides the *kdr* mutation, our study suggested the involvement of other resistance mechanisms of *An. gambiae* to pyrethroids in the region of Plateau.

Based on the resistance criteria we defined, more than 70% of the 56 prospected localities were classified as R^+++^ localities. This confirms the rapid expansion of the resistance phenomenon across the region. Because of this expansion, we were not able to find the eight susceptibility localities for the implementation of our impact study of the resistance of *An. gambiae* on the effectiveness of LLINs. Instead, we had to define R^+^ and R^+++^ areas for the implementation of our study.

## Competing interests

The authors declare that they have no competing interests.

## Authors’ contributions

AS, ID, AM, MA and VC have participated in the design of the study. ID, LS, AS and FT carried out the field activities and the laboratory analyses. VG has contributed to the mapping. AS and MA drafted the manuscript. FOA contributed to the statistical analysis. MO, RA, AA, ID, AM, FT and VC critically revised the manuscript for intellectual content. All authors read and approved the final manuscript.

## Pre-publication history

The pre-publication history for this paper can be accessed here:

http://www.biomedcentral.com/1471-2334/14/103/prepub

## References

[B1] Ministère de la Santé du Bénin, Programme National de Lutte contre le Paludisme Evaluation par la méthode LQAS de la campagne intégrée d’octobre 2007 de distribution des MIILD, de l’Albendazole et de la vitamine A aux enfants de moins de cinq ans et du niveau de quelques indicateurs de suivi de la lutte contre le paludisme 2009 Cotonou, Benin: PNLP

[B2] Elissa N Mouchet J Rivière F Meunier JY Yao K Resistance of *Anopheles gambiae* s.s. to pyrethroids in Côte d’Ivoire Ann Soc Belge Med Trop 1993 14 291 294 8129474

[B3] Akogbéto M Yacoubou S Resistance of Malaria vectors to pyrethroids used for impregnated bednets, Benin, West Africa Bull Soc Path Exo 1999 14 123 130 10399604

[B4] Chandre F Manguin S Brengues J Dossou YJ Darriet F Diabate A Carnevale P Guillet P Current distribution of pyrethroid resistance gene (kdr) in *Anopheles gambiae* complex from West Africa and further evidence for reproductive isolation of Mopti form Parasitologia 1999 14 319 322 10697876

[B5] Yadouleton AW Padonou G Asidi A Moiroux N Bio-Banganna S Corbel V N'Guessan R Gbenou D Yacoubou I Gazard K Massougbodji A Akogbéto M Insecticide resistance status in *Anopheles gambiae* in southern Benin Malar J 2010 14 83 10.1186/1475-2875-9-83 20334637 PMC2858214

[B6] Stump AD Atieli FK Vulule JM Besansky NJ Dynamics of the pyrethroid knockdown resistance allele in Western Kenya populations of *Anopheles gambiae* in response to Insecticide-treated bed net trial Am J Trop Med Hyg 2004 14 591 596 15210997

[B7] Etang J Fonjo E Chandre F Morlais I Brengues C Nwane P Chouaibou M Ndjemai H Simard F Short report: first report of knockdown mutations in the malaria vector *Anopheles gambiae *from Cameroon Am J Trop Med Hyg 2006 14 795 797 16687682

[B8] Hargreaves K Koekemoer L Brooke BD Hunt RH Mthembu J Coetzee M *Anopheles funestus* resistant to pyrethroid insecticides in South Africa Med Vet Entomol 2000 14 181 189 10.1046/j.1365-2915.2000.00234.x 10872862

[B9] N'Guessan R Corbel V Akogbeto M Rowland M Reduced efficacy of insecticide-treated nets and indoor residual spraying for malaria control in pyrethroid resistance area, Benin Emerg Infect Dis 2007 14 199 206 10.3201/eid1302.060631 17479880 PMC2725864

[B10] Institut National de la Statistique Appliquée et de l’Economie Recensement général de la population du Bénin 2002 Cotonou, Benin: INSAE

[B11] O'Malley C Seven ways to a successful dipping career Wing Beats 1995 14 4 23 24

[B12] WHO Report of the informal consultation. Test procedures for insecticide resistance monitoring in malaria vectors, bio efficacy and persistence of insecticides on treated surfaces 1998 Geneva, Switzerland: World Health Organization: Parasitic Diseases and Vector Control (PVC)/Communicable Disease Control, Prevention and Eradication (CPE)

[B13] Scott J Brogdon W Collins F Identification of single specimens of the *Anopheles gambiae* complex by PCR Am J Trop Med Hyg 1993 14 520 529 8214283 10.4269/ajtmh.1993.49.520

[B14] Favia G Della TA Bagayoko M Lanfrancotti A Sagnon NF Toure Y Coluzzi M Molecular identification of sympatric chromosomal forms of *Anopheles gambiae* and futher evidence of their reproductive isolation Insect Mol Biol 1997 14 377 383 10.1046/j.1365-2583.1997.00189.x 9359579

[B15] Lynd A Ranson H McCall PJ Randle NP Black WC Walker ED Donnelly MJ A simplified high-throughput method for pyrethroid Knock-down resistance (*kdr*) detection in *Anopheles gambiae* Malar J 2005 14 16 10.1186/1475-2875-4-16 15766386 PMC555548

[B16] Dobson AJ An Introduction to Generalized Linear Models, Second Edition 2001 London: Chapman & Hall/CRC

[B17] Chambers JM Hastie TJ Linear models. Chapter 4 of Statistical Models in S 1992 Pacific Grove, California: Wadsworth & Brooks/Cole

[B18] R Development Core Team A language and environment for statistical computing 2011 Vienna, Austria: R Foundation for Statistical Computing http://www.r-project.org. ISBN ISBN 3-900051-07-

[B19] Padonou G Sezonlin M Ossé R Aizoun N Oké-Agbo F Oussou O Gbédjissi G Akogbéto M impact of three years of large scale Indoor Residual Spraying (IRS) and Insecticide Treated Nets (ITNs) interventions on insecticide resistance in Anopheles gambiae s.l. in Benin Parasit Vectors 2012 14 72 10.1186/1756-3305-5-72 22490146 PMC3379941

[B20] Ranson H N’Guessan R Lines J Moiroux N Nkuni Z Corbel V Pyrethroid resistance in African anopheline mosquitoes: what are the implications for malaria control? Trends Parasitol 2011 14 2 91 98 10.1016/j.pt.2010.08.004 20843745

[B21] Corbel V N’Guessan R Brengues C Chandre F Djogbenou L Martin T Akogbeto M Hougard JM Rowland M Multiple insecticide resistance mechanisms in *Anopheles gambiae* and *Culex quinquefasciatus* from Benin, West Africa Acta Trop 2007 14 207 216 10.1016/j.actatropica.2007.01.005 17359927

[B22] Djogbenou L Noel V Agnew P Costs of insensitive acetylcholinesterase insecticide resistance for the malaria vector *Anopheles gambiae* homozygous for the G119S mutation Malar J 2010 14 12 10.1186/1475-2875-9-12 20070891 PMC2816975

[B23] Djegbe I Boussari O Sidick A Thibaud M Ranson H Chandre F Akogbeto M Corbel V Dynamics of insecticide resistance in malaria vectors in Benin: first evidence of the presence of *L1014S* kdr mutation in *Anopheles gambiae* from West Africa Malar J 2011 14 261 10.1186/1475-2875-10-261 21910856 PMC3179749

[B24] Protopopoff N Verhaeghen K Van Bortel W Roelants P Marcotty T Baza D D’Alessandro U Coosemans M A high increase in kdr in *Anopheles gambiae* is associated with an intensive vector control intervention in Burundi highlands Trop Med Int Health 2008 14 1479 1487 10.1111/j.1365-3156.2008.02164.x 18983277

[B25] Czeher C Labbo R Arzika I Duchemin JB Evidence of increasing Leu-Phe knockdown resistance mutation in *Anopheles gambiae* from Niger following a nationwide long-lasting insecticide-treated nets implementation Malar J 2008 14 189 10.1186/1475-2875-7-189 18817574 PMC2562389

[B26] Yadouleton A Thibaud M Padonou G Chandre F Asidi A Djogbenou L Dabiré R Aïkpon R Boko M Glitho I Akogbeto M Cotton pest management practices and the selection of pyrethroid resistance in Anopheles gambiae population in Northern Benin Parasit & Vect 2011 14 60 10.1186/1756-3305-4-60 PMC3082239 21489266

[B27] Yadouleton AW Asidi A Djouaka RF Braïma J Agossou CD Akogbeto MC Development of vegetable farming: a cause of the emergence of insecticide resistance in populations of *Anopheles gambiae* in urban areas of Benin Malar J 2009 14 103 10.1186/1475-2875-8-103 19442297 PMC2686728

[B28] Akogbéto M Romano R Infectivité d'*Anopheles melas* vis-à-vis du *Plasmodium falciparum* dans le milieu côtier lagunaire du Bénin Bull Soc Pathol Exot Filiales 1999 14 57 61 10214525

[B29] Costantini C Ayala D Guelbeogo WM Pombi M Some CY Bassole IH Ose K Fotsing JM Sagnon N Fontenille D Besansky NJ Simard F Living at the edge: biogeographic patterns of habitat segregation conform to speciation by niche expansion in *Anopheles gambiae* BMC Ecol 2009 14 16 10.1186/1472-6785-9-16 19460144 PMC2702294

[B30] Djènontin A Bio-Bangana S Moiroux N Henry MC Boussari O Chabi J Ossè R Koudénoukpo S Corbel V Akogbéto M Chandre F Culicidae diversity, malaria transmission and insecticide resistance alleles in malaria vectors in Ouidah-Kpomasse-Tori district from Benin (West Africa): a pre-intervention study Parasites & Vect 2010 14 83 10.1186/1756-3305-3-83 PMC2941749 20819214

[B31] Diabaté A Baldet T Chandre F Akogbéto M Guiguendé TR Darriet F Brengues C Guillet P Hemingway J Small GJ Hougard JM The role of agricultural use of insecticides in resistance to pyrethroids in *Anopheles gambiae s.l.* in Burkina Faso Am J Trop Med Hyg 2002 14 6 617 622 12518852 10.4269/ajtmh.2002.67.617

[B32] Dabiré KR Diabaté A Djogbenou L Ouari A N'Guessan R Ouédraogo JB Hougard JM Chandre F Baldet T Dynamics of multiple insecticide resistance in the malaria vector *Anopheles gambiae* in a rice growing area in South-Western Burkina Faso Malar J 2008 14 188 10.1186/1475-2875-7-188 18817564 PMC2564969

[B33] Dabiré KR Diabaté A Agostinho F Alves F Manga L Faye O Baldet T Distribution of the members of *Anopheles gambiae* and pyrethroid knock-down resistance gene (*kdr*) in Guinea-Bissau, West Africa Bull Soc Pathol Exot2008b 14 101 119 123 18543705

[B34] Ranson H Jensen B Vulule JM Wang X Hemingway J Collins FH Identification of a point mutation in the voltage-gated sodium channel gene of Kenyan *Anopheles gambiae* associated with resistance to DDT and pyrethroids Insect Mol Biol 2000 14 491 497 10.1046/j.1365-2583.2000.00209.x 11029667

[B35] Müller P Chouaïbou M Pignatelli P Etang J Walker ED Donnelly MJ Simard F Ranson H Pyrethroid tolerance is associated with elevated expression of antioxidants and agricultural practice in *Anopheles arabiensis* sampled from an area of cotton fields in Northern Cameroon Mol Ecol 2008 14 1145 1155 18179425 10.1111/j.1365-294X.2007.03617.x

[B36] Djouaka RF Bakare AA Coulibaly ON Akogbeto MC Ranson H Hemingway J Strode C Expression of the cytochrome P450s, CYP6P3 and CYP6M2 are significantly elevated in multiple pyrethroid resistant populations of Anopheles gambiae s.s. from Southern Benin and Nigeria BMC Genomics 2008 14 538 10.1186/1471-2164-9-538 19014539 PMC2588609

[B37] Djouaka R Irving H Tukur Z Wondji SC Exploring Mechanisms of Multiple Insecticide Resistance in a Population of the Malaria Vector *Anopheles funestus* in Benin PLos one 2011 14 e27760 10.1371/journal.pone.0027760 22110757 PMC3218031

[B38] Tokponnon FT Aholoukpe B Denon EY Gnanguenon V Bokossa A N’guessan R Oke M Gazard D Akogbeto MC Evaluation of the coverage and effective use rate of long-lasting insecticidal nets after nation-wide scale up of their distribution in Benin Parasites & Vectors 2013 14 265 10.1186/1756-3305-6-265 24499613 PMC3848614

